# Liquid‐Metal Interfacial Chemistry for Crystal and Defect Engineering in Two‐Dimensional Post‐Transition‐Metal Materials

**DOI:** 10.1002/advs.77978

**Published:** 2026-09-27

**Authors:** Zhejun Hong, Mohammad B. Ghasemian, Chung Kim Nguyen, Huy Hoang Nguyen, Kourosh Kalantar‐Zadeh, Torben Daeneke, Nitu Syed, Ali Zavabeti

**Affiliations:** ^1^ Department of Chemical and Environmental Engineering RMIT University Melbourne Victoria Australia; ^2^ School of Chemical and Biomolecular Engineering The University of Sydney Darlington New South Wales Australia; ^3^ Centre of Excellence for Future Assistive Technologies Faculty of Natural Sciences and Technology Riga Technical University Riga Latvia

**Keywords:** alloy, crystal, defect engineering, doping, interfacial engineering, interfacial reaction, liquid metal, materials science

## Abstract

Liquid‐metal‐derived 2D materials have emerged as a versatile class of ultrathin systems enabled by dynamic and chemically active liquid metal interfaces. The atomically smooth, self‐limiting surface layers formed at these interfaces provide unique pathways for the synthesis and transfer of ultrathin post‐transition metal materials, supporting applications in optoelectronics, sensing, energy, and catalysis. However, material formation at liquid metal interfaces is governed by complex interfacial chemistry involving redox reactions, alloy thermodynamics, atomic migration, reactive atmospheres, and processing conditions. These processes directly influence crystal formation, phase, stoichiometry, and the generation of point, planar, and morphological defects. Here, we review how liquid metal interfaces control crystal and defect formation in 2D materials, highlighting key factors that influence defect formation, including atmospheric conditions, alloy composition, doping, and post‐processing. We also discuss how these defects influence their electronic, optical, sensing, and piezoelectric properties. By reframing defects as tunable design parameters rather than unavoidable limitations, this Review establishes liquid metal interfacial chemistry as a powerful framework for engineering functional 2D materials with tailored properties and improved technological performance.

## Graphical abstract

Liquid‐metal interfaces provide dynamic reaction environments in which crystal growth, composition, and defect formation are intrinsically coupled. This Review reveals how interfacial oxidation, alloy thermodynamics, elemental migration, atmosphere, and processing govern defects in 2D post‐transition‐metal materials, and how these defects can be engineered to enable sensing, electronics, optoelectronics, catalysis, electromechanical functionality, and neuromorphic devices.

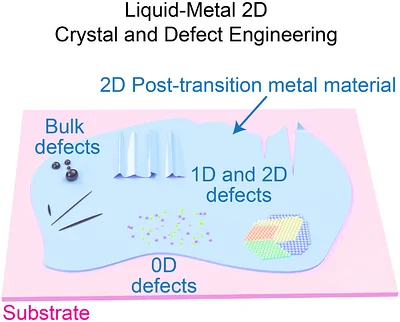

## 1 Introduction

The remarkable properties of 2D materials are governed by their crystal structure, thickness, and defect chemistry [1]. Reduced dimensionality and high surface‐to‐volume ratios give rise to growth mechanisms that differ fundamentally from bulk systems, controlled by thermodynamic and kinetic factors such as atomic diffusion, interfacial bonding, and substrate interactions [2, 3]. These processes not only define crystallinity but also inevitably generate defects, which are intrinsic to ultrathin materials [4, 5]. Rather than being passive imperfections, defects actively modulate structural, electronic, optical, and chemical properties [6, 7]. This dual role was first highlighted in graphene, where defects reduce carrier mobility yet introduce chemical reactivity and enable band structure engineering [8].

**FIGURE 1:**
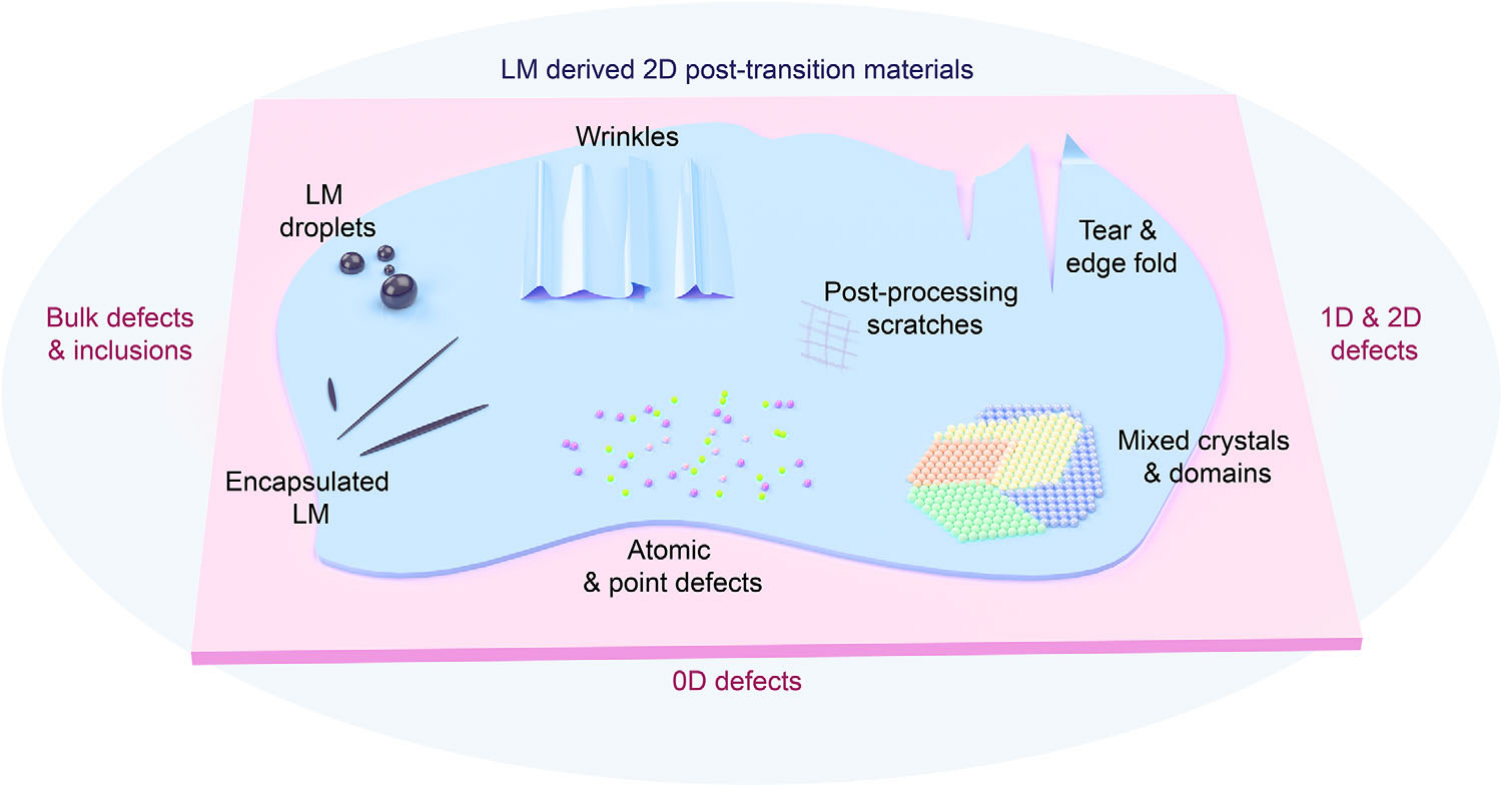
Schematic illustration of inherent defects in liquid‐metal‐derived 2D materials, spanning bulk, 2D, 1D, and 0D regimes, originating from liquid metal dynamics, manual transfer and processing, and atomic‐scale growth mechanisms.

Liquid‐metal (LM) derived 2D post‐transition materials have emerged as a distinct class, where interfacial redox reactions at liquid–gas boundaries enable the scalable formation of ultrathin oxides and mixed systems with tunable composition, structure, and functionality [9]. However, these advantages are intrinsically accompanied by a rich diversity of structural defects arising from three simultaneously operating processes: (i) interfacial redox reactions, including oxidation, and related chemical transformations that define nucleation pathways and stoichiometry, (ii) fluidic deformation and surface renewal of the liquid metal that continuously disrupt and regenerate the growth interface, and (iii) mechanical and chemical perturbations during exfoliation and post‐growth film transfer. (Figure 1). As a result, defect formation in LM‐derived 2D materials is not incidental but fundamentally linked to their synthesis pathways. Defect chemistry in LM‐derived 2D materials is therefore unavoidable but not inherently detrimental. Depending on their type and density, defects may act as scattering centers and cause instability, or conversely, they may serve as active sites that enhance gas adsorption, catalytic activity, or charge separation [10, 11, 12, 13, 14, 15]. Reported examples include point defects such as vacancies, substitutional dopants, planar defects like grain boundaries, and morphological defects such as wrinkles and folds [9, 13, 14, 16, 17, 18]. The energetic stability and structural expression of these defects are strongly influenced by the kinetics of LM oxidation and exfoliation, making them highly tunable but also highly variable.

Accordingly, studying defect chemistry in LM‐derived 2D materials is both a scientific necessity and a technological opportunity. From a fundamental standpoint, it provides insight into how atomic‐scale disorder governs emergent properties in low‐dimensional systems. From an applied perspective, it offers practical pathways to intentionally exploit or mitigate defects for applications in sensing, catalysis, optoelectronics, and energy conversion. This review, therefore, aims to summarize the origins, classifications, and characterization of defects in LM‐derived 2D systems, and to discuss strategies for their controlled chemistry to unlock new functionalities and device performance. Overall, defect engineering requires simultaneous control over interfacial chemistry, fluid dynamics, and transfer mechanics. Recognizing this coupled regime provides a unifying basis to interpret the diverse defect structures reported across LM‐derived systems and, more importantly, establishes a pathway toward predictive and programmable defect design.

## 2 Interfacial Chemistry for Atomically Thin Materials Growth in Liquid Metals

Liquid metals provide a unique platform for interfacial chemistry, enabling the synthesis of atomically thin functional materials through spontaneous surface oxidation and coupled interfacial reactions [19].

### 2.1 Interfacial Oxidation Dynamics of Liquid Metal

**FIGURE 2:**
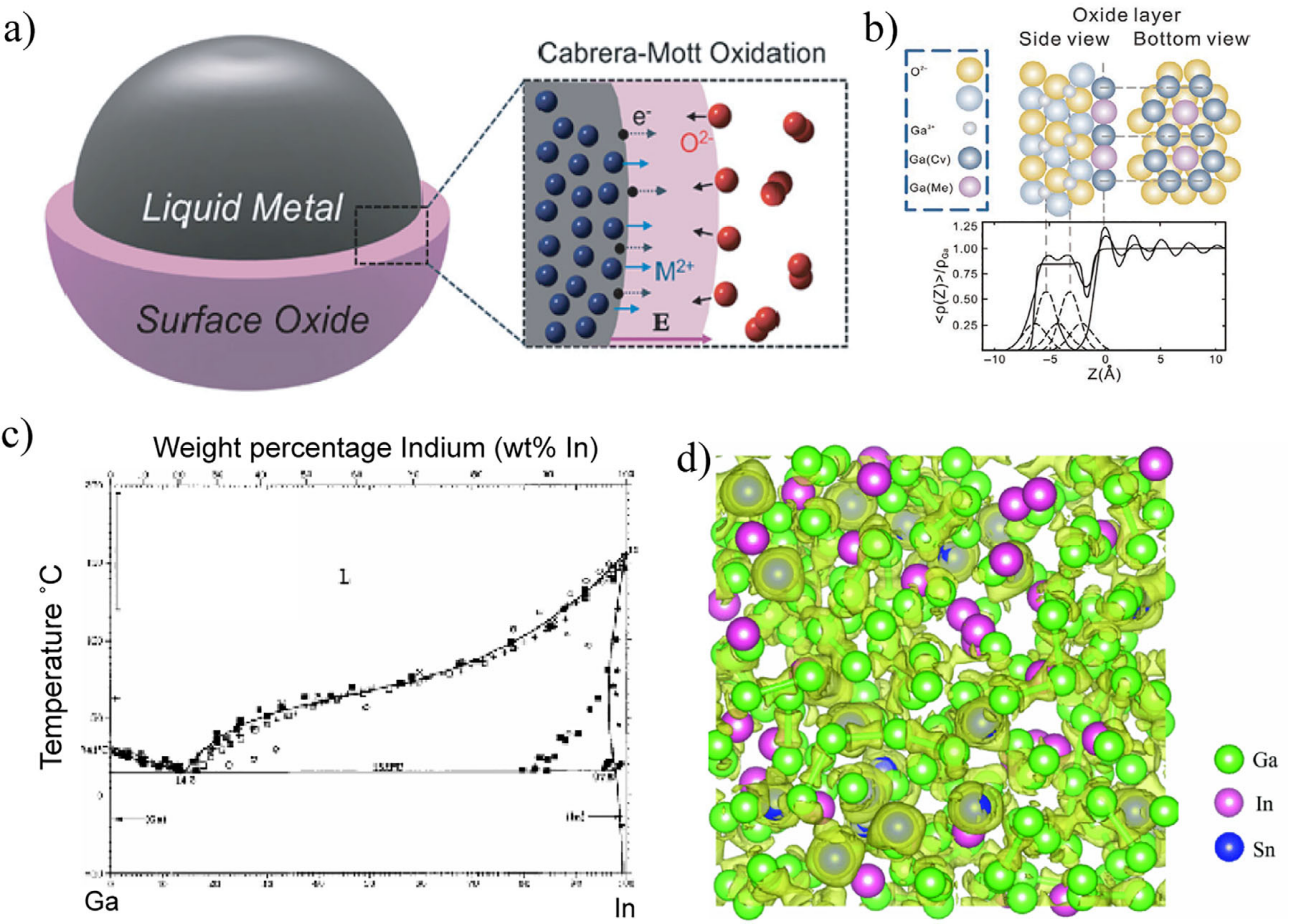
Growth chemistry on LM‐gas interface. (a) Schematic illustration of the Cabrera–Mott oxidation mechanism for surface oxide formation on LM, reproduced with permission [20, 33]. Copyright 2024, Wiley‐VCH. (b) Model for the proposed atomic arrangement in the oxide skin of Ga and the corresponding electron density profile. Atomic diameters: O^2−^: 2.64 Å, Ga^3+^: 1.24 Å, Ga (Cv: covalent): 2.44 Å, Ga (Me: metal): 2.50 Å, reproduced with permission [34]. Copyright 1997, American Physical Society. (c) Phase diagram of the binary Ga–In system. Various data points were experimentally determined, reproduced with permission [24]. Copyright 2007, Springer Nature. (d) Atomic distributions inside eutectic GaInSn determined via density functional theory (DFT) calculations, reproduced with permission [35]. Copyright 2014, American Institute of Physics.

At the liquid metal interface, ultrathin oxide layers form spontaneously upon exposure to oxygen, even at very low concentrations (>1 ppm). Oxygen molecules first adsorb onto the liquid metal surface, followed by electron transfer from the metal to the adsorbed oxygen species (Figure 2a,b). This charge transfer establishes a potential difference across the oxide layer (often referred to as the Mott potential), generating a strong electric field that drives oxidation [20]. The growth of this oxide layer is governed by the Cabrera–Mott oxidation mechanism, which describes field‐assisted ion transport through ultrathin films. The rate of oxide growth can be expressed as [21]:

(1) $$\frac{dX}{dt}=N^{\prime }\;\Omega v\cdot \exp\left(\frac{-W}{kT}\right)\cdot \exp\left(\frac{\;qa^{\prime }F}{kT}\right)\;$$

The oxidation kinetics of liquid metals can be described in terms of ionic transport through the growing oxide layer, where the growth rate depends on oxide thickness (*X*), surface density of mobile ions (N′), oxide volume per ion (Ω), and atomic vibrational frequency (ν). The activation barrier *W*  = *W_i_*  + *U*includes both the heat of solution and diffusion energy. Physically, oxidation is governed by thermally activated ion migration and the electric field across the oxide. Increasing temperature enhances ion mobility, while the internal electric field lowers the effective energy barrier and drives ion transport. Under strong‐field conditions, particularly for ultrathin films, oxidation proceeds rapidly before transitioning to a self‐limiting regime as the oxide thickens [21].

Beyond this intrinsic behavior, alloying provides an additional level of control over oxide formation. Multicomponent liquid metals form low‐melting eutectic systems (e.g., Ga–In, Ga–In–Sn), enabling facile processing (Figure 2c,d) [22, 23, 24]. Oxidation in these systems is thermodynamically driven, where elements with more negative Gibbs free energy preferentially oxidize at the interface [25, 26]. Subsequent interfacial diffusion enables dopant incorporation and mixed‐oxide formation [26, 27, 28, 29, 30]. This selective oxidation governs defect structure and composition, as demonstrated in Zn–Sn systems, ultimately tuning electronic and catalytic properties [31].

From a defect‐formation perspective, the oxidation stage plays a central role in determining the formation and distribution of atomic‐scale defects within the native oxide. Variations in environmental temperature, atmospheric composition, and ionic transport kinetics can drive deviations from ideal stoichiometry and influence the concentration of native point defects, such as oxygen vacancies [32]. In multicomponent liquid metals, differences in elemental oxygen affinity and interfacial transport can further introduce local variations in oxide composition and stoichiometry [31]. Consequently, interfacial oxidation governs not only the thickness and composition of the resulting 2D oxide, but also the formation and distribution of its intrinsic defects.

### 2.2 Interfacial Printing of Atomically Thin Materials From Liquid Metals

**FIGURE 3:**
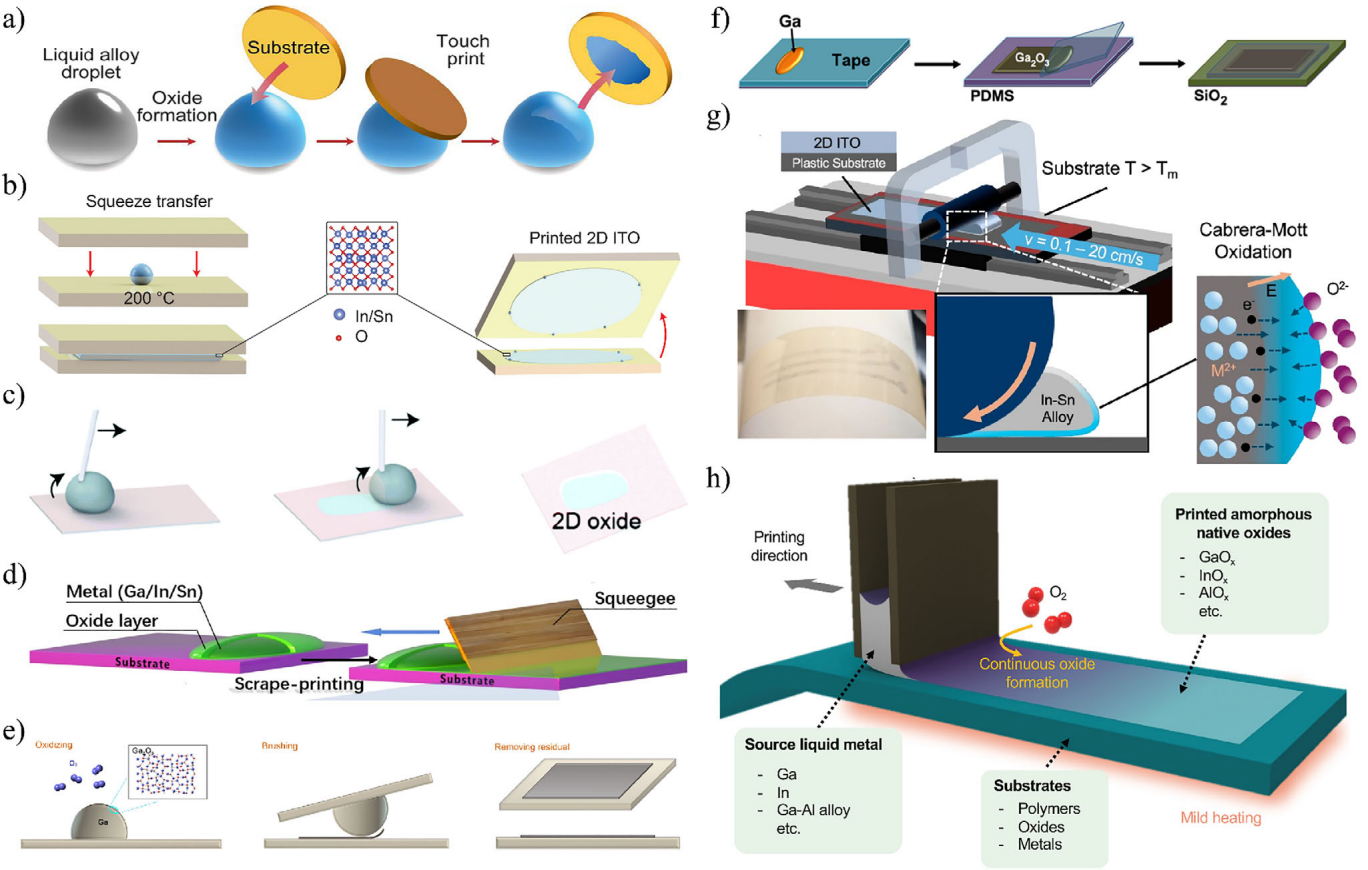
(a) Touch printing, reproduced with permission [9]. Copyright 2017, Science (AAAS). (b) Squeeze printing, reproduced with permission [39]. Copyright 2020, Springer Nature. (c) Roll printing, reproduced with permission [25]. Copyright 2022, Royal Society of Chemistry. (d) Blade‐coating, reproduced with permission [42]. Copyright 2019, American Chemical Society. (e) Brush printing, reproduced with permission [43]. Copyright 2020, American Chemical Society. (f) Printing using PDMS‐assisted transfer, reproduced with permission [44]. Copyright 2024, Springer Nature. (g) Kinetically controlled LM printing, reproduced with permission [45]. Copyright 2024, Springer Nature. (h) Dewetting‐induced printing, reproduced with permission [46]. Copyright 2024, Science (AAAS).

Van der Waals (vdW) exfoliation and touch printing of 2D metal oxides (MOs) from a liquid metal platform were first reported in 2017 (Figure 3a) [9, 25, 36, 37]. Weak interfacial interactions between the oxide skin and the underlying liquid metal enable facile physical exfoliation, analogous to graphene isolation via vdW forces [9, 38]. This provides a simple route to atomically thin oxide layers without complex processing. Squeeze printing extends this approach by applying gentle pressure to confine a liquid metal droplet between substrates, producing larger‐area oxide films (Figure 3b) [25, 39]. During deformation, the liquid metal continuously reforms fresh oxide skin through rapid oxidation, healing defects and enabling uniform film transfer. This method has also been adapted to synthesize doped oxides, including indium tin oxide and antimony‐doped systems, demonstrating its versatility for scalable fabrication of functional 2D materials [39, 40]. Roll printing enables controlled deposition of 2D metal oxides by guiding a eutectic Se‐Te liquid metal droplet across a substrate (Figure 3c), yielding extended and uniform films [41]. Related approaches, including blade coating (Figure 3d) and brush printing (Figure 3e), rely on dynamic interfacial contact and continuous oxide regeneration during droplet motion to produce conformal layers [36, 42, 43]. These methods provide improved control over film continuity and lateral dimensions compared with static transfer techniques. Further integration with tape‐assisted transfer and elastomeric substrates such as PDMS (Figure 3f) enables the fabrication of large‐area, continuous films with smooth surfaces and thicknesses of a few nanometers [44]. Collectively, these strategies highlight the scalability of liquid metal‐mediated 2D oxide printing.

Scalability and film uniformity remain key challenges, limiting the industrial translation of LM‐derived 2D metal oxides (MOs). To address this, Rahman et al. introduced a substrate‐driven rolling approach, in which a liquid metal droplet is pressed by a silicone roller and translated across the substrate at controlled speeds (0.1–20 cm s^−^^1^), producing homogeneous films over areas exceeding 100 cm^2^ (Figure 3g) [45]. Although this kinetically controlled method enables large‐area fabrication, residual liquid metal necessitates post‐processing. Dewetting‐induced printing also offers an alternative strategy to minimize such residues. In this approach, a confined liquid metal meniscus between vertically aligned glass slides collapses via dewetting as the print head moves, depositing uniform bilayer oxide films (Figure 3h) [46]. Despite sub‐10 nm thickness and high cleanliness, achieving optimal film quality requires precise control of printing parameters. Together, these advances highlight promising pathways toward scalable, high‐quality 2D MO manufacturing.

When synthesizing 2D oxides using liquid metals, their versatility extends to non‐oxide 2D materials through controlled precursor chemistry and reaction environments [47]. For example, exposure to H_2_S yields 2D sulfides, while post‐synthetic treatments such as sulfidation or nitridation convert LM‐derived oxides into diverse ultrathin materials beyond oxides [48, 49, 50]. Other representative examples include the growth of uniform graphene flakes and films on liquid Cu, onto Ga using organic precursors and electrophoretic placement on a substrate, as well as the synthesis of ultrathin nitrides using liquid metal‐assisted precursor transport and in a two‐step process upon printing and annealing with urea [49, 51, 52, 53]. These studies, together with recent developments in liquid metal‐mediated atomic engineering, demonstrate that liquid interfaces provide various routes to chalcogenides, nitrides, carbon‐based materials, and related 2D systems, where precursor supply, diffusion, and interfacial kinetics provide additional opportunities for controlling structure and defects [47, 52, 53, 54]. Furthermore, liquid metals can also function as redox‐active substrates, where precursor compounds are reduced at the LM interface to form ultrathin 2D layers [55, 56, 57].

Although printing techniques enable thin oxide deposition on diverse substrates, the printing and transfer steps can introduce defects distinct from those generated during interfacial oxidation, making precise defect control challenging. Whereas oxidation primarily influences atomic‐scale point defects and stoichiometric deviations within the oxide lattice, the hydrodynamic, mechanical, and interfacial forces involved in printing and transfer tend to generate larger‐scale structural defects. These include wrinkles, folds, cracks, local thickness variations, discontinuities, and residual or encapsulated liquid‐metal droplets. Residual liquid metal often requires post‐transfer cleaning, which may further introduce defects, such as surface damage and thickness inhomogeneities.

Importantly, oxidation‐induced and printing‐induced defects are not completely independent. Printing and transfer processes can modify the conditions under which the interfacial oxide forms or regenerates, thereby influencing oxidation kinetics and the subsequent formation and distribution of defects. For example, variations in environmental temperature, atmospheric composition, liquid‐metal surface state, interfacial contact time, mechanical stress, and local wetting conditions can affect ionic transport and oxide‐growth dynamics. Mechanical rupture of the native oxide during printing can also expose fresh liquid‐metal surfaces, leading to repeated oxidation and oxide regeneration. Therefore, the final defect structure of printed 2D oxides often reflects the coupled effects of interfacial oxidation and the subsequent printing and transfer processes.

## 3 Defect Formation and Evolution in Liquid‐Metal‐Derived Crystal Growth

The crystal growth of 2D MOs from liquid metals is inherently dynamic, involving rapid surface oxidation, exfoliation, printing, and transfer processes. While these approaches enable scalable synthesis of ultrathin films, they also introduce a variety of defects that strongly influence the microstructure and functionality of the resulting materials.

For clarity, defects in LM‐derived 2D materials can be broadly classified according to their stage of formation into growth‐induced, interfacial, and post‐processing defects, as summarized in Table 1.

**TABLE 1:** Classification of defects in liquid‐metal‐derived 2D materials according to their formation stage.

Formation stage	Dominant origin	Typical defects	Characteristic scale	References
Interfacial oxidation and film formation	Field‐assisted ion transport, oxidation thermodynamics/kinetics, nucleation and crystallization	Vacancies and interstitials, non‐stoichiometry, compositional fluctuations, grain boundaries	Atomic/nanoscale	[21, 32, 58, 59, 60, 61, 62]
Printing and transfer	Hydrodynamic, mechanical and interfacial forces, oxide rupture, repeated oxidation	Wrinkles, cracks, folds, voids, discontinuities, thickness variations, LM residues	Nano–micro/larger scale	[36, 39, 42, 45, 63, 64, 65, 66]
Post‐processing (cleaning and annealing)	Mechanical and interfacial forces, oxidation/reduction and recrystallization	Surface damage, contamination, defect redistribution and healing, vacancies, grain‐boundary evolution	Multiscale	[17, 18, 25, 39, 49, 67, 68, 69]

From a thermodynamic standpoint, perfect structural order is only strictly attainable at absolute zero. At any finite temperature (*T* > 0) entropy introduces configurational disorder, rendering point‐defect formation thermodynamically accessible. The equilibrium defect concentration follows Boltzmann statistics [70, 71]:

(2) $$C_{Defect}\left(T\right)\propto e^{-\frac{E_{f}}{k_{B}T}}$$

where *C_Defect_* (*T*) is the defect density, *E_f_* is the defect formation energy, *k_B_* is the Boltzmann constant, and *T*is the synthesis temperature in Kelvin. Equation (2) reflects the probability of thermally activated access to higher‐energy configurations relative to the ideal lattice. In a more generalized description, multiple point‐defect types contribute to the overall disorder:

(3) $$C_{Total}\left(T\right)\propto \sum_{j}A_{j}e^{-\frac{E_{j}}{k_{B}T}}$$

where *E_j_* and *A_j_* correspond to the formation energy and prefactor associated with defect species *j*, including vacancies, interstitials, antisites, and other atomic structural disorders [70, 71]. Equations (2) and (3) provide an idealized thermodynamic framework rather than a quantitative prediction of defect populations in LM‐derived 2D materials. In practice, defect concentrations and distributions also depend on oxidation kinetics, atmosphere and oxygen availability, alloy composition, elemental diffusion and interfacial transport, and printing or transfer conditions.

In an ideal crystal, perfect translational symmetry ensures that all atoms occupy well‐defined lattice positions without impurities. In practice, however, LM‐derived 2D oxides inevitably contain defects whose nature and origin depend strongly on the stage at which they are generated (Table 1). Temperature also activates competing kinetic processes that promote structural reordering and defect annihilation. Processes such as diffusion, elimination of vacancies, grain boundary migration and coarsening, and recrystallisation become increasingly efficient at elevated temperatures or during post‐growth annealing [4, 5, 7, 72]. These competing processes can also be described by a general Arrhenius‐type recovery rate [73]:

(4) $$R\left(T\right)\propto e^{-\frac{E_{a}}{k_{B}T}}$$

where *R*(*T*)is the defect annihilation or recovery rate and *E_a_* is the activation energy associated with defect migration or healing. Equation (4) reflects the thermally activated nature of annealing processes, whereby increased atomic mobility enables defects to redistribute, recombine, or be eliminated, leading to improved crystallinity and larger grain sizes. This behavior is widely established in materials science, particularly in the context of recovery and recrystallisation phenomena during annealing.

Overall, the defect population in LM‐derived 2D oxides reflects a competition between thermodynamically accessible defect formation (Equations (2), (3)) and kinetically activated defect migration, redistribution, and annihilation (Equation 4), as further discussed in later sections. The interaction among these factors ultimately determines the electronic and functional properties of liquid‐metal‐derived 2D oxides.

### 3.1 Atomic‐Scale Point and Planar Defects

**FIGURE 4:**
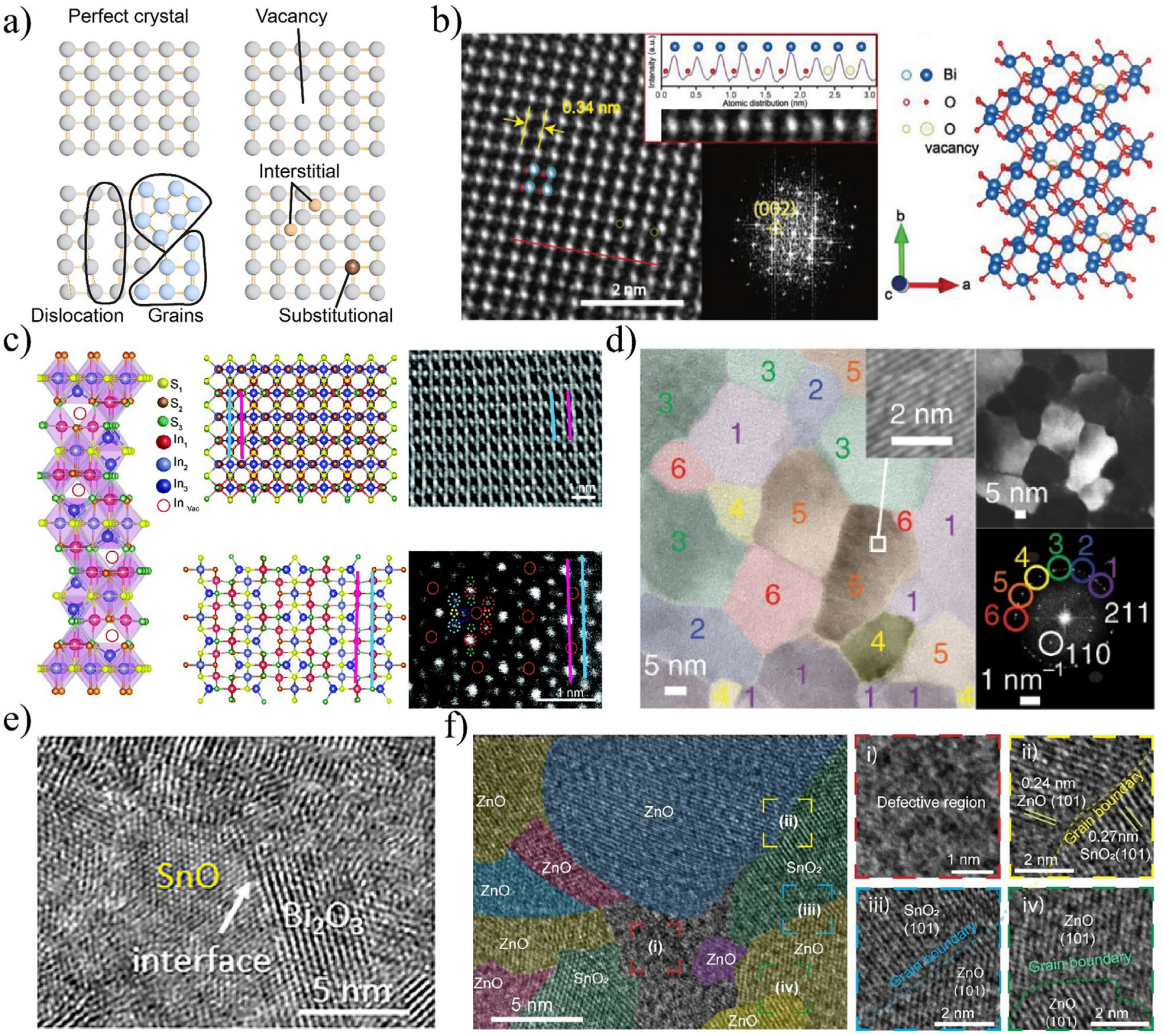
(a) Ideal crystal and typical defects. (b) HRTEM image of 𝛼‐Bi_2_O_3−δ_. An example signal intensity profile along the red line is shown, reproduced under terms of the CC‐BY license [74]. Copyright 2023, Wiley‐VCH. (c) Ordered‐vacancy‐enabled indium sulfide printed on a wafer scale, and the crystal schematic of a single unit cell of tetragonal In_2_S_3_, reproduced with permission [75]. Copyright 2020, Royal Society of Chemistry. (d) HRTEM image of the optimized 2D ITO nanosheet. The color code highlights the crystal orientation based on the fast Fourier transform (FFT) image shown in the lower right inset. The upper left inset shows a magnified view of the lattice pattern, and the upper right image shows a dark‐field image of the region of interest, reproduced with permission [39]. Copyright 2020, Springer Nature. (e) HRTEM image of Bi_2_O_3_‐doped SnO nanosheet harvested from the surface of 90% Bi liquid metal, presenting SnO and Bi_2_O_3_ phases and their interfaces, reproduced with permission [76]. Copyright 2021, Wiley‐VCH. (f) HRTEM image with color‐coded areas illustrating different crystal grains of both ZnO and SnO_2_ within the nanosheet, (i–iv) selected zoom‐in areas indicating the presence of defective regions and grain boundaries, reproduced under the terms of the CC‐BY license [31]. Copyright 2025, Wiley‐VCH.

Point defects arise from missing, displaced, or substituted atoms as shown in Figure 4a [11]. In LM‐derived oxides, vacancies (particularly oxygen vacancies) form readily during surface oxidation of Ga, In, or Sn [14, 32, 75]. These defects act as donor states, increasing carrier density and enhancing conductivity, while also serving as active sites for gas adsorption. Interstitial defects, formed when atoms occupy non‐lattice positions under high‐energy conditions such as rapid oxidation, introduce local lattice distortion and strain [11, 77]. Antisite defects, in which atoms exchange lattice positions, are less common in 2D oxides due to their relatively high formation energies compared to vacancies and interstitials [78]. Impurity defects, either substitutional or interstitial, further modulate electronic properties depending on their valence state [11]. Experimental studies highlight the functional role of such defects. For instance, oxygen vacancies in LM‐derived Bi_2_O_3_ enable enhanced piezotronics responses (Figure 4b) [74]. Similarly, liquid‐metal‐printed In_2_O_3_ converted to In_2_S_3_ exhibits an ordered‐vacancy tetragonal structure (Figure 4c), promoting efficient electron transport through extended conduction pathways [75, 79]. Thus, point defects in LM‐derived oxides are not merely unavoidable but serve as tunable electronic and catalytic features that can be engineered via alloying, annealing, or controlled oxidation.

Planar defects, including grain boundaries, stacking faults, and phase interfaces, are 2D irregularities that strongly influence 2D oxide properties [17, 31, 39, 80]. Grain boundaries form during coalescence of oxide domains and, while reducing carrier mobility, provide chemically active sites beneficial for catalysis and sensing (Figure 4d) [81, 82]. Phase boundaries in mixed or doped oxides, such as In–Sn, Zn–Sn, or Sn–Bi systems, arise from preferential oxidation and modulate defect density and electronic behavior (Figure 4e,f) [39, 76]. Owing to their surface accessibility, planar defects act both as transport barriers and functional sites in 2D materials.

### 3.2 Bulk Defects and Morphological Defects

**FIGURE 5:**
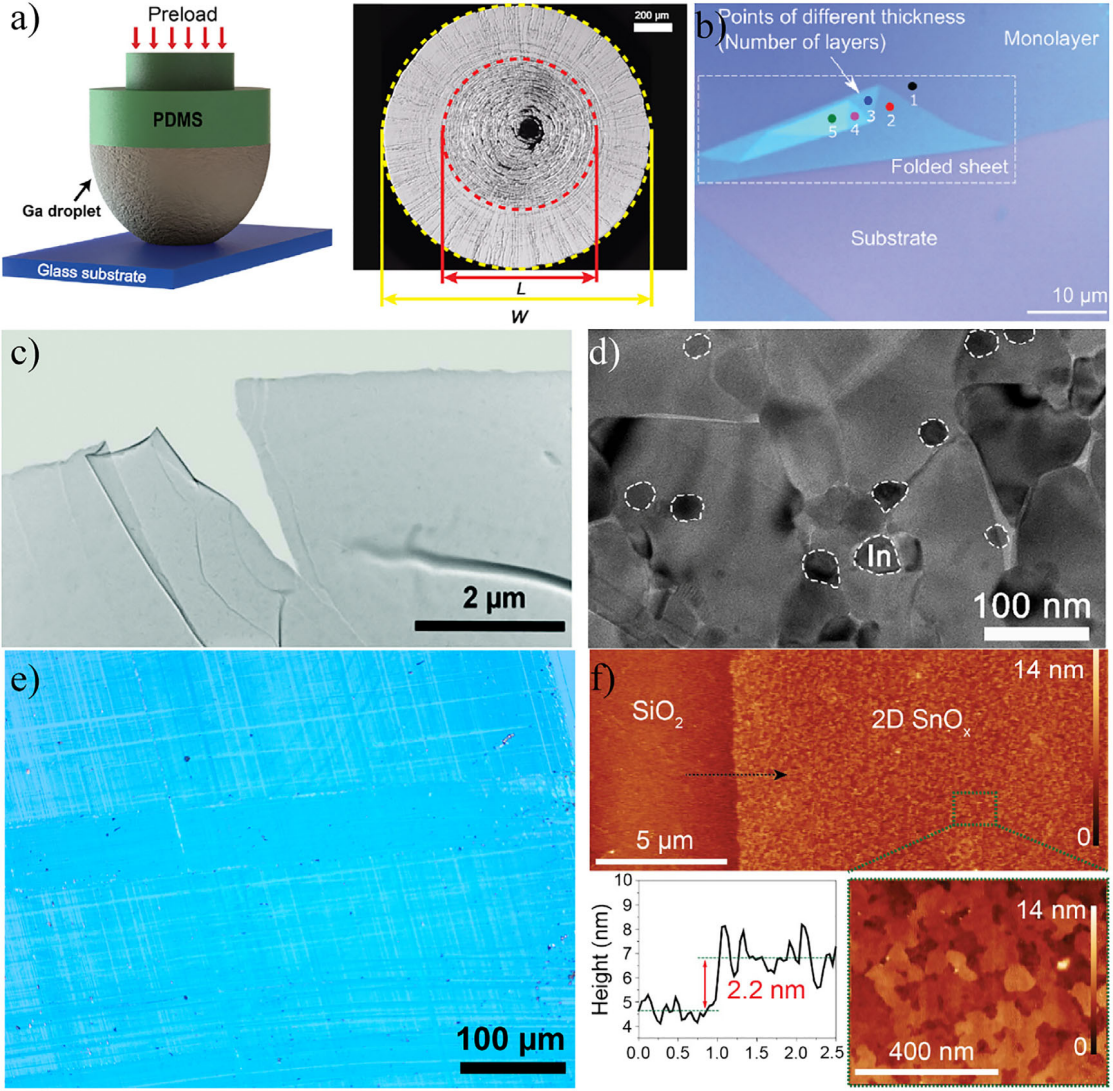
(a) Liquid metal droplet and printed film with morphological defects due to the flattening process, reproduced with permission [83]. Copyright 2018, American Chemical Society. (b) Optical microscopy image of the 2D morphology synthesized on Liquid Metals with folding edges, reproduced with permission [56]. Copyright 2020, Wiley‐VCH. (c) Folds, cracks and wrinkles, reproduced with permission [84]. Copyright 2018, Royal Society of Chemistry. (d) Top view TEM image of the In‐embedded ultrathin In_2_O_3_ films before annealing, reproduced with permission [85]. Copyright 2025, Wiley‐VCH. (e) Scratches and dust on Ga_2_O_3_ due to an improper cleaning process. (f) AFM images of ultrathin SnO*_x_* nanosheets show fish‐scale‐like morphological defects, reproduced with permission [18]. Copyright 2024, American Chemical Society.

Bulk defects in LM‐derived 2D oxides arise from mechanical stresses during printing and transfer, manifesting as wrinkles, folds, cracks, pores, and thickness variations. Although often classified as planar in 2D systems, these features occur at larger length scales and strongly influence device performance. Wrinkles and folds, originating from the convex geometry of liquid metal droplets and weak oxide adhesion (Figure 5a), generate local strain fields that shift band edges and modify adsorption energies. Thickness inhomogeneities also arise during droplet spreading, particularly at overlapping edges [9].

Additional defects, including cracks, pores, folds, and inclusions formed during exfoliation (Figure 5b–d), and scratches introduced during cleaning (Figure 5e), disrupt electrical pathways and reduce film uniformity. Such defects can be mitigated using controlled printing strategies, including piezo‐assisted stages and automated processes [46, 76]. Morphological features may also emerge during growth. For example, “fish scale‐like” structures in SnO_x_ nanosheets reflect thickness variations and can be reduced through annealing (Figure 5f) [18]. Overall, these defects represent both challenges and opportunities, linking growth dynamics with functional performance and enabling defect engineering for sensing, catalysis, and optoelectronics.

### 3.3 Defects in Mixed Crystal Structure and Substitutional Impurities

Foreign atoms can also be incorporated into the lattice of 2D materials as substitutional impurities. Substitutional dopants are expected to be very stable due to strong covalent bonding. Mixed crystal structures are an intrinsic feature of LM‐derived oxides formed from multicomponent alloys. Lattice mismatch between constituent phases generates additional grain boundaries, planar defects, and vacancies, which significantly influence material properties. As shown in Figure 4f, these defect‐rich regions provide abundant adsorption sites for gas molecules [31]. For example, Zn–Sn alloys at eutectic composition produce mixed ZnO‐SnO_2_ films, where lattice mismatch induces oxygen vacancies and enhances surface reactivity, improving sensing performance [13, 14]. Preferential oxidation further governs phase formation; metals with more negative Gibbs free energy of oxide formation, such as Zn, oxidize more readily, driving selective oxide growth [86, 87].

Doping also promotes mixed crystal formation when dopant atoms integrate into the host lattice, forming solid solutions or defect‐stabilized phases [16, 88, 89]. In LM systems, selective oxidation of multicomponent alloys often leads to mixed crystals rather than isolated dopants, owing to differences in oxygen affinity and diffusion kinetics. High‐entropy liquid metals provide a notable example, where multiple elements (In, Pt, Au, Pd, Cu) are incorporated into gallium to form ultrathin oxide films with tunable electronic properties [90]. Noble metals facilitate oxygen exchange, stabilizing specific oxidation states and enabling p‐type behavior in Ga_2_O_3_ with enhanced conductivity and transparency [90, 91, 92]. Doping concentration further influences structure and growth dynamics. For instance, antimony incorporation in indium oxide alters ionic transport and increases oxide thickness by modifying diffusion kinetics at the liquid metal–air interface [40, 93]. Overall, mixed crystal formation in LM‐derived oxides reflects a complex interplay between alloy composition, interfacial oxidation, and defect chemistry, offering a powerful route to tune functionality.

## 4 Factors Governing Crystal Structures and Defect Formation

The crystal structure and defect landscape of liquid‐metal‐derived ultrathin materials are governed by a complex interplay of thermodynamic and kinetic factors. The synthesis of the few‐atom‐thick metal oxides (MOs) typically involves oxidation to form an interfacial oxide skin, exfoliation onto a substrate, removal of residual liquid metal, and post‐processing. Key parameters, including alloy composition, oxidation kinetics, temperature, and interfacial dynamics, dictate atomic arrangement, phase stability, and defect density [25]. Understanding these factors is essential for controlling mixed‐crystal formation and tailoring functional properties.

**FIGURE 6:**
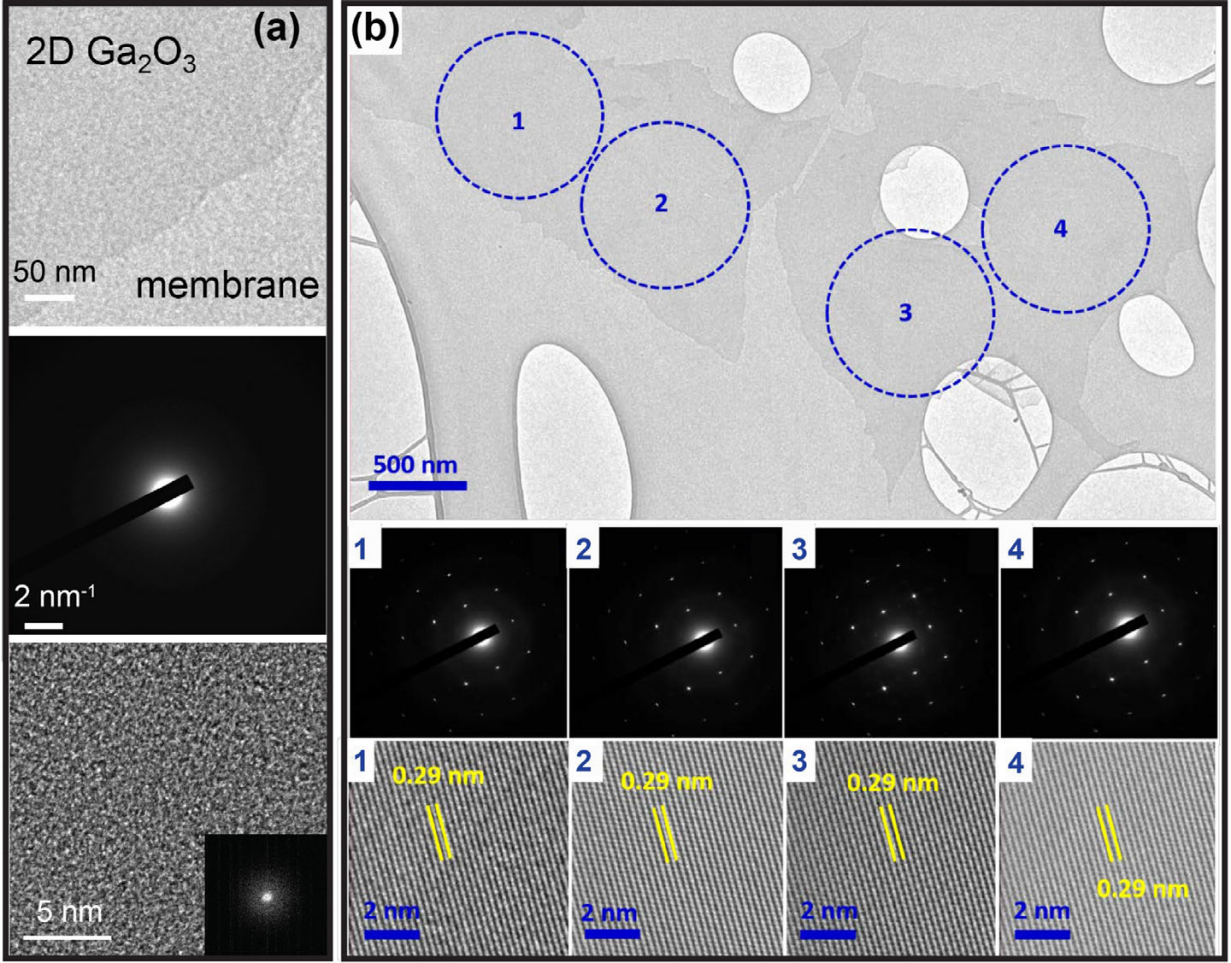
From amorphous to single‐crystal growth governed by temperature and environmental control. (a) Ga_2_O_3_ synthesized near room temperature under ambient conditions (air with moisture), yielding amorphous structures, as evidenced by SAED, HRTEM and FFT analyses showing an absence of long‐range crystallinity, reproduced with permission [94]. Copyright 2020, Wiley‐VCH. (b) Liquid‐metal‐derived 2D SnS synthesized under stringent environmental control (extended inert gas purging) and elevated temperature, producing large‐area single‐crystalline domains. Representative regions demonstrate highly ordered lattice alignment, confirmed by SAED and HRTEM consistent with single‐crystal characteristics [48].

Gallium oxide exemplifies LM‐derived systems formed near room temperature, where films are often amorphous (Figure 6a) [94]. Ambient exposure introduces moisture‐driven hydroxide formation (e.g., GaOOH, Ga(OH)_3_), increasing structural complexity and defect formation. In contrast, synthesis under control or elevated‐temperature conditions (>100°C) suppresses moisture effects and enhances crystallinity. A pressure‐assisted liquid‐metal printing technique has been reported, enabling the low‐temperature synthesis of polycrystalline wide‐bandgap n‐channel oxide [95]. Liquid‐metal‐derived SnS grown under controlled environments yields near single‐crystalline monolayers with minimal defects (Figure 6b) [48]. Together, these observations establish temperature and environmental conditions as key parameters governing crystallinity and defect populations in LM‐derived materials. Given these profound effects, the roles of temperature and environment are discussed in the following section.

### 4.1 Synthesis Temperature

Oxidation at the liquid metal surface underpins the formation of 2D metal oxides and critically determines film quality. Temperature is a key parameter governing this process. For instance, the thickness of liquid‐metal‐printed indium oxide increases from ∼4 nm at 170°C to ∼16 nm at 300°C, with low‐temperature films remaining largely amorphous, while higher temperatures promote crystallization and grain growth [32]. Initially, oxide formation follows the Cabrera–Mott regime, where electron tunnelling drives growth; however, once a critical thickness is reached, further growth is limited and transitions to thermally activated diffusion (Wagner regime) [21]. Subsequent annealing enhances recrystallisation, reduces grain boundaries, and improves crystallinity [59].

**FIGURE 7:**
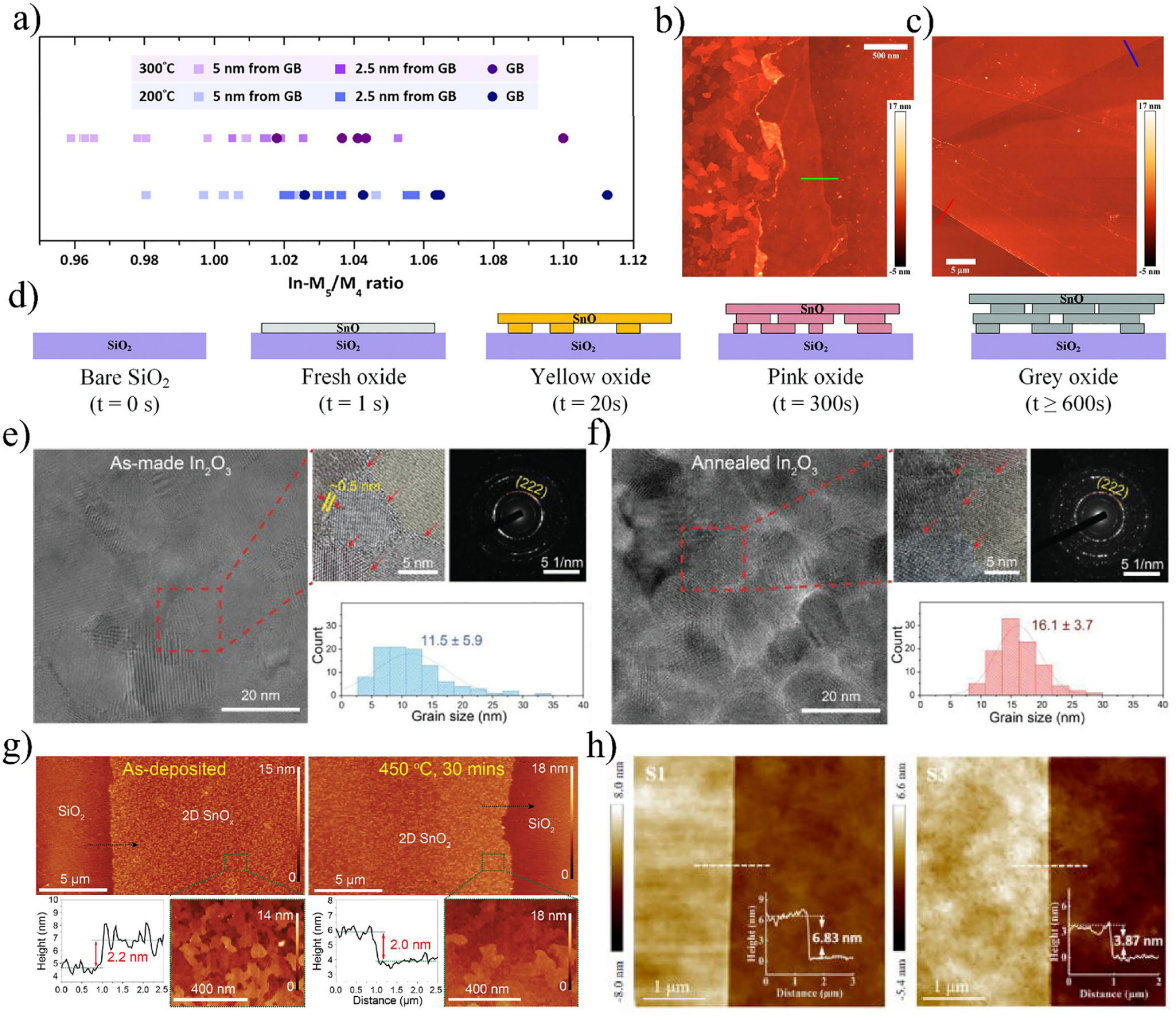
(a) In‐M_5_/In‐M_4_ ratios, reproduced under terms of the CC‐BY license [32]. Copyright 2024, American Chemical Society. (b) AFM image of the tin oxide synthesized under an ambient atmosphere, and (c) tin oxide synthesized in a continuously purged glovebox (N_2_ atmosphere, O_2_ concentration typically between 10 and 100 ppm), reproduced with permission [62]. Copyright 2017, American Chemical Society. (d) SnO_x_ obtained at varying intervals (t) following preconditioning, reproduced with permission [61]. Copyright 2018, Royal Society of Chemistry. (e) HRTEM images with corresponding zoom‐in areas, SAED patterns, and grain size distributions of pristine and (f) annealed 2D In_2_O_3_ nanosheets, reproduced with permission [17]. Copyright 2023, Wiley‐VCH. (g) AFM images of SnO*_x_* nanosheets at different annealing conditions with the step height profiles (lower left insets) and zoomed‐in regions (lower right insets), reproduced with permission [18]. Copyright 2024, American Chemical Society. (h) AFM images of 2D‐Ga_2_O_3_ films, corresponding to an as‐made sample (50°C) and an annealed sample (800°C for 1 h), where the fresh sample showed a thickness of ∼6.83 nm and the annealed one showed thickness reduction to 3.87 nm due to enhanced film‐substrate adhesion, recrystallization, and atomic rearrangement, reproduced with permission [69]. Copyright 2026, American Institute of Physics.

Temperature also strongly influences defect formation. Figure 7a shows In‐M_5_/In‐M_4_ ratios obtained from several grain boundaries and grain interior regions, while the In‐M_5_/In‐M_4_ ratio determination reflects the variation in valence states of In cations. The larger ratio of 200°C 2D‐InO*_x_* signifies a greater reduction in the valence state of indium attributable to the formation of oxygen vacancies and suggests that low‐temperature printing results in a higher concentration of defects in 2D‐InO*_x_* [21, 32, 58]. The observed decrease in defect concentration at higher printing temperatures can be attributed to enhanced atomic diffusion, which promotes defect migration and annihilation, leading to a lower concentration of oxygen vacancies [21, 58].

Lower temperatures favor the retention of oxygen vacancies, whereas higher temperatures reduce defect density through enhanced atomic diffusion and reduced lattice distortion energy [32, 96]. Thermal annealing further promotes recrystallisation and grain growth, improving structural order. In addition, substrate temperature affects droplet spreading, fluid dynamics, and film uniformity, influencing thickness distribution and microstructure [63, 64]. Overall, temperature acts as a central control parameter that couples oxidation kinetics with defect evolution, ultimately determining the nanostructure and morphology of LM‐derived 2D oxides.

### 4.2 Atmospheric Control

Tuning oxidation conditions, including exposure time, atmospheric composition, and oxygen partial pressure, enables control over the thickness, phase, and crystallinity of liquid‐metal‐derived oxides. For example, gallium forms a self‐limiting Ga_2_O_3_ layer under ambient conditions, where growth is governed by oxygen availability and diffusion barriers [60]. In contrast, metals such as tin exhibit more complex oxidation behavior, forming multiple oxide phases depending on oxygen concentration [61, 62]. Under ambient conditions, SnO_x_ sheets with a SnO_2_ surface layer are typically formed (Figure 7b), whereas oxygen‐reduced environments favor uniform monolayer SnO formation (Figure 7c). Further control of oxygen exposure enables precise tuning of the oxide thickness and stoichiometry (Figure 7d) [61, 62].

### 4.3 Interfacial Dynamics, Exfoliation Strategies and Sample Preparation

Beyond oxidation control, the quality of liquid‐metal‐derived (LM) oxides is strongly influenced by film separation and transfer. Detaching the ultrathin oxide layer from the liquid–gas interface and transferring it onto a substrate is inherently delicate, with mechanical stress, adhesion mismatch, and interfacial energy gradients often introducing defects such as cracks, wrinkles, and voids. Manual transfer methods, including touch printing onto substrates or TEM grids, are particularly susceptible to operator‐dependent disturbances, resulting in structural inhomogeneity, non‐uniform coverage, and reduced reproducibility [65]. Various transfer strategies have therefore been developed to mitigate these issues. Squeeze printing reduces cracking through rapid self‐healing oxidation but can introduce thickness variations and interfacial boundaries [49]. In contrast, scraper‐ or roller‐assisted methods enable more uniform, large‐area films, although residual droplets may oxidize and generate localized defects [36, 42, 45]. More controlled approaches, such as piezo‐assisted positioning, can further improve transfer reproducibility and minimize transfer‐induced artifacts.

From a practical standpoint, residual LM inclusions must be carefully controlled, as they can interact with metallic components, particularly aluminum, potentially causing structural degradation and damage to instrumentation through scratching or chemical interaction. This is especially critical during transfer and characterization, where Ga‐based LM can interact with metallic electrodes such as gold, altering both electrode integrity and local composition. As a result, LM‐based device fabrication often relies on post‐deposition patterning rather than direct integration. However, photolithographic steps can introduce contamination or modify ultrathin layers, necessitating alternative strategies such as hard‐mask patterning, albeit with limitations in resolution and device architecture [41]. Notably, confined liquid metal droplets and encapsulated micro‐layers can exhibit pronounced supercooling behavior, remaining in a metastable liquid state tens of degrees below their bulk melting point [97, 98]. As a result, nanoscale droplet inclusions and rolled or encapsulated liquid domains within LM‐derived 2D sheets may persist in a liquid or semi‐liquid state, acting as sources of local disorder, strain, plasmonic response, and electrical inhomogeneity.

The substrate also plays a critical role, with its surface energy, lattice structure, and thermal properties governing adhesion, crystal alignment, and stress evolution, thereby influencing defect formation and overall film quality [66]. Poor interfacial compatibility can promote incomplete adhesion, local delamination, strain accumulation, and nonuniform film morphology. Residual or encapsulated liquid‐metal inclusions may also remain after transfer, creating local structural and compositional heterogeneity. For example, oxygen‐terminated surfaces can promote adhesion and lateral continuity, whereas non‐oxide (e.g., Si) or metallic substrates may exhibit reduced adhesion, resulting in fragmented nanosheets and limited large‐area coverage. These observations highlight the importance of interfacial energetics, surface chemistry, and lattice mismatch, which remain insufficiently explored in LM‐derived systems. Substrate engineering, including surface functionalization and thermal matching, can further suppress defect formation and improve film uniformity. Consequently, optimizing substrate–film interactions is essential for achieving reproducible, high‐quality 2D oxide devices.

### 4.4 Post‐Processing Factors

Post‐processing steps, particularly cleaning and annealing, play a critical role in defining the defect landscape and crystal structure of liquid‐metal‐derived ultrathin materials. During exfoliation and transfer, residues from the liquid metal or transfer media can remain on the surface, introducing extrinsic defects and modifying surface chemistry. Cleaning treatments including solvent rinsing, mild etching, or plasma exposure are therefore indispensable for preparing high‐quality 2D metal oxides. A widely adopted approach involves gently wiping excess liquid metal using cotton swabs in warm ethanol or octanol [25, 39, 49, 67]. However, this manual process can introduce bulk defects, such as scratches and contamination from residual liquid metal (Figure 5e). Minimizing applied pressure, reducing wiping cycles, and using softer fibers are essential to mitigate such damage. Recent studies have shown that under controlled interfacial conditions, the native oxide layer can be delaminated as a continuous ultrathin film with minimal residue, thereby avoiding mechanical damage associated with conventional cleaning methods. However, such residue‐free transfer is highly sensitive to wetting behavior and peeling dynamics, and is not universally achieved across all substrates [99].

Annealing further governs defect evolution and phase stability by enabling atomic rearrangement, recrystallisation, and defect healing [17, 18, 68]. Its effectiveness depends on temperature, duration, and atmosphere. For example, annealing In_2_O_3_ at ∼250°C improves transistor performance by shifting operation modes and enhancing carrier mobility through nano‐structural reorganization [68]. Structural analysis reveals that annealing enlarges crystal domains (Figure 7f), reduces grain boundaries, and absorbs intergranular distortions present in as‐prepared films (Figure 7e), thereby improving charge transport [17]. Similar improvements are observed in SnO_x_ systems, where annealing at 450°C modifies phase composition and reduces defect states (Figure 7g) [18]. In Ga_2_O_3_, annealing enables tunable lattice ordering and bandgap modulation (Figure 7h), with thickness reduction linked to enhanced adhesion and recrystallisation [69]. Overall, post‐processing provides a powerful route to tailor defect density, crystallinity, and functional performance, although optimization must be carefully adapted to specific materials and applications.

### 4.5 Defect Characterization in Liquid‐Metal‐Derived 2D Materials

Accurate identification of defects in liquid‐metal‐derived 2D materials requires complementary structural, chemical, and spectroscopic characterization techniques. Defects can occur across multiple length scales, ranging from atomic‐scale vacancies and compositional fluctuations to grain boundaries, wrinkles, cracks, and thickness nonuniformities. Accordingly, no single characterization technique can provide a complete description of the defect landscape. Moreover, characterization itself can introduce artifacts, particularly in ultrathin and beam‐sensitive materials, and these effects must be carefully distinguished from intrinsic or processing‐induced defects. Electron microscopy techniques, including high‐resolution transmission electron microscopy (HRTEM) and aberration‐corrected transmission electron microscopy (AC‐TEM), provide atomic‐scale insight into lattice structure, grain boundaries, and defect configurations, although limited sampling areas and complex sample preparation restrict statistical analysis [76]. Integration with electron energy loss spectroscopy (EELS) enables spatial mapping of electronic structure, where low‐loss plasmon features reveal localized inhomogeneities in disordered or high‐entropy systems, particularly relevant for LM‐derived multi‐component oxides [90, 100].

X‐ray photoelectron spectroscopy (XPS) probes chemical states but suffers from limited spatial resolution and surface‐dominated signals due to rapid oxidation in LM systems [36]. Importantly, XPS mapping can provide enhanced spatial resolution for detecting compositional inhomogeneity; for example, dispersed species such as Se within TeO_2_ matrices can be resolved through lateral mapping [41]. In contrast, optical spectroscopic techniques such as Raman spectroscopy and photoluminescence (PL) offer rapid, non‐destructive, and large‐area characterization, providing insights into crystal structure, lattice vibrations, strain, thickness, charge transfer, and defect‐induced disorder [101].

Scanning probe techniques further complement these approaches. Atomic force microscopy (AFM) provides morphology and thickness information, while scanning tunnelling microscopy/spectroscopy (STM/STS) can probe local electronic states, including mid‐gap states [102]. Conductive AFM (c‐AFM) provides spatially resolved measurements of local conductivity and can be used to identify electrically active defects [102, 103]. Despite these advances, defect analysis remains challenging because of processing‐induced artefacts, environmental exposure, and dynamic interfacial chemistry. A comparative summary of the information accessible through the principal characterization techniques and their major limitations is provided in Table 2. These challenges highlight the need for integrated and in situ characterization strategies.

**TABLE 2:** Comparison of representative techniques for defect characterization in liquid‐metal‐derived 2D materials.

Characterization technique	Main information obtained	Main limitations	References
HRTEM/AC‐TEM	Atomic structure, vacancies, grain boundaries, lattice defects, atom identification	Small sampling area, complex preparation, electron‐beam‐induced damage or structural modification	[76]
EELS	Elemental and low‐loss region features distribution, plasmon, band gap, bonding, oxidation state, local electronic structure.	Beam sensitivity, complex interpretation, usually TEM‐dependent	[90, 100]
XPS/UPS	Composition, oxidation state, surface chemistry, stoichiometry	Surface‐sensitive, limited spatial resolution, peak‐fitting ambiguity	[36, 41]
Raman/PL/Time resolved PL	Crystal structure, lattice vibrations, strain, disorder, thickness, charge transfer, recombination dynamics and carrier/exciton lifetime	Indirect defect assignment, weak signals in ultrathin materials	[101]
AFM	Morphology, roughness, thickness, wrinkles and cracks	Limited chemical and crystallographic information	[102]
STM/c‐AFM/PFM/KPFM	Local electronic states including trap states, mid‐gap, and bandgap spectroscopy and conductivity variations, surface potential/contact potential difference and charge trapping, local electromechanical response, polarization/symmetry breaking and effective piezoresponse	Small scan area and tip–sample/contact effects; require suitable conductive pathways; PFM can be affected by electrostatic, capacitive and contact‐mechanical artefacts; KPFM is sensitive to tip calibration, surface charging and environmental conditions	[103, 104]
Atom probe tomography (APT)	Emerging method for 3D elemental distribution, atomic‐scale compositional depth profiling, dopant/impurity segregation and interface chemistry	Destructive; demanding needle‐shaped specimen preparation; very small analysis volume; reconstruction and field‐evaporation artefacts	[105]
ToF‐SIMS	Elemental/molecular composition, high‐resolution depth profiling, compositional gradients and buried interlayers in ultrathin films	Destructive sputtering; matrix effects; limited absolute quantification; sputter‐induced mixing may affect depth resolution	[46]
X‐ray reflectometry (XRR)	Film thickness, electron‐density/depth profiles, layer density, interface structure and buried interlayers, useful for identifying compositional or density gradients through ultrathin films	Model‐dependent fitting; limited direct chemical specificity; provides laterally averaged information and generally requires relatively smooth, uniform films.	[46]
Synchrotron XAS (XANES/EXAFS)	Element‐specific oxidation state, local coordination environment, bond lengths, coordination number, local structural disorder, and dopant–host bonding, useful for determining how defect/substitutional/adatoms modify the local crystal environment	Requires synchrotron access; data analysis and fitting can be model‐dependent, provides an ensemble‐averaged local structure rather than direct spatial imaging, rarely investigated in 2D LM materials.	[46, 106]

## 5 Emerging Applications Enabled by Defects and Mixed‐Crystal Structures

Defect chemistry in liquid metal‐derived 2D materials underpins a wide range of multifunctional applications, including gas sensing, electronics, optoelectronics, piezotronics systems, and neuromorphic devices. The impact of defects on device performance depends not only on their density but also on their type, charge state, spatial distribution, and interaction with the surrounding lattice. Point defects such as oxygen vacancies and dopants can modify carrier concentration, introduce localized electronic states, alter adsorption energetics, and promote ionic transport. Grain boundaries can provide chemically active sites while regulating intergrain carrier transport. Heterointerfaces can modulate charge transport, polarization, and carrier dynamics. These effects can influence sensitivity, responsivity, switching voltage, carrier mobility, piezoelectric output, response speed, and long‐term stability of the final devices. Controlled defect incorporation, including vacancy ordering and dopant engineering, enables optimization of electronic transport and extension of carrier lifetimes. However, excessive defect densities can introduce deep trap states and degrade performance, highlighting the need for precise defect control [12, 13, 14, 31, 40, 48, 50, 74, 75, 107, 108, 109, 110, 111, 112, 113, 114, 115, 116, 117, 118].

### 5.1 Gas Sensing

Defects in liquid metal–derived oxides can strongly enhance sensing while also influencing piezoelectric behavior. Oxygen vacancies, dopants, and mixed‐valence states create active sites and promote charge transfer, improving gas adsorption, sensitivity, and response dynamics. At the same time, defects can distort the local crystal structure and alter charge distribution, directly affecting polarization and piezoelectric response.

In gas‐sensing applications, oxygen vacancies influence the response through both surface chemistry and electronic transport [119, 120]. In many semiconducting metal oxides, oxygen vacancies modify the electronic structure and increase the density of active surface sites, thereby enhancing chemisorption and influencing gas‐sensing performance [119, 120]. Adsorbed oxygen species can capture electrons from the oxide, inducing surface band bending and an electron‐depletion region, while exposure to reducing gases can alter this surface charge state through reactions with the adsorbed oxygen species. Consequently, defect‐mediated adsorption is translated into a measurable change in conductivity [121]. Increasing the oxygen‐vacancy concentration can enhance gas‐sensing performance by increasing the number of active surface sites, enhancing chemisorption, and modifying the electrical conductivity of the material [119, 120]. Grain boundaries provide a second mechanism for tuning sensing performance, which can provide additional adsorption sites for gas molecules, while grain‐boundary potential barriers can regulate carrier transport between adjacent crystalline domains [31, 122]. Adsorption at grain boundaries can modify these potential barriers and thereby change the film conductivity. In this way, a small amount of adsorbed species at grain boundaries can produce a significant sensing response [123]. This coupling between adsorption and intergrain transport can therefore enhance sensitivity, although grain boundaries can also reduce carrier mobility by acting as charge‐carrier scattering centers [31, 123].

**FIGURE 8:**
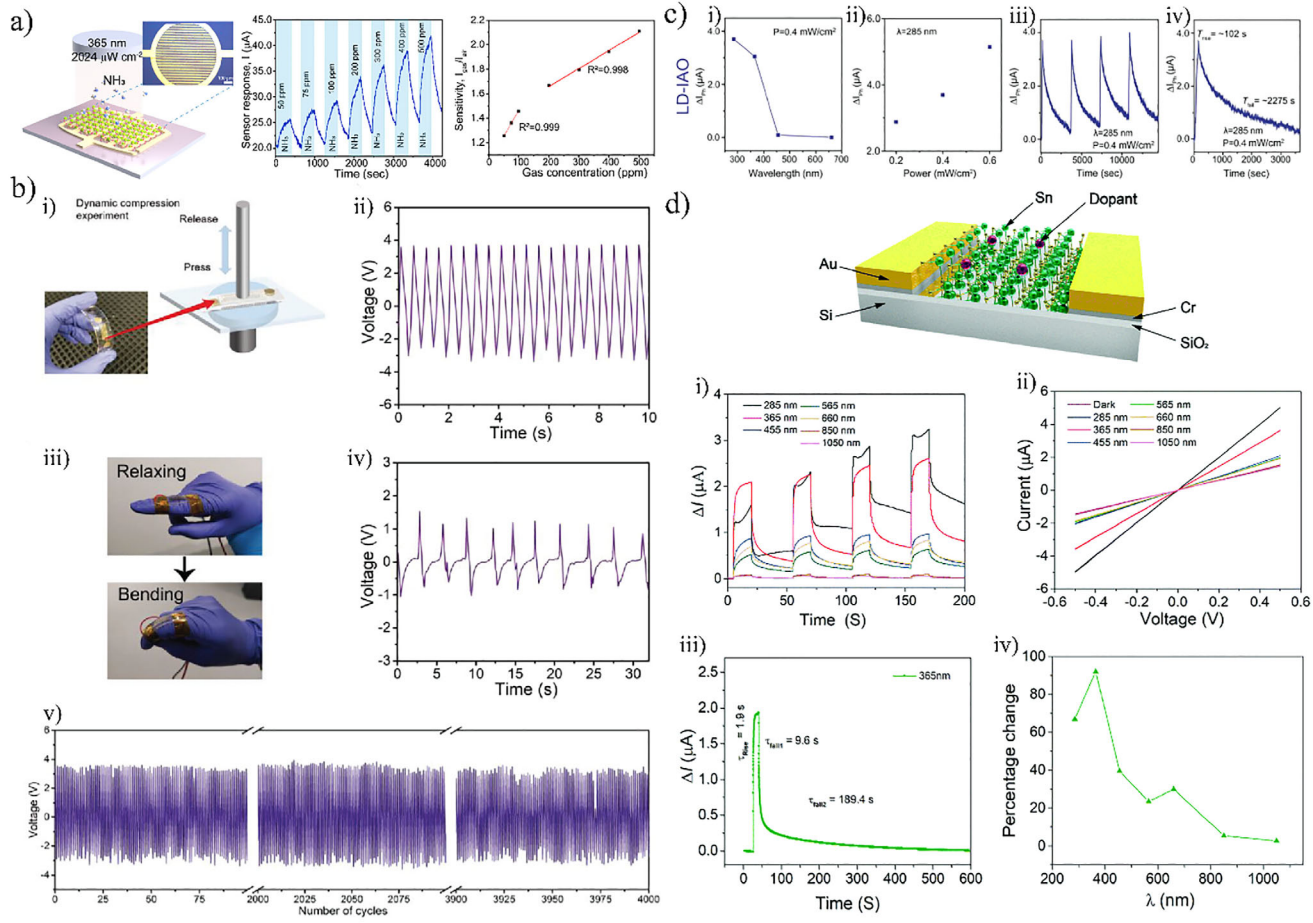
(a) The optical image of the IDE sensor fabricated on ZTCMO nanosheets, along with its response profile of different concentrations of NH_3_ and sensitivity of the sensor to different NH_3_ concentrations from 50 to 500 ppm at room temperature, reproduced under terms of the CC‐BY license [31]. Copyright 2025, Wiley‐VCH. (b) (i) The photo of a representative nanogenerator device. (ii) Response of the output voltage of the device under vertical pressing. (iii) Signal acquisition of the bending behavior of the flexible devices attached to finger joints. (iv) Voltage output acting on stretch‐bend and relaxation. (v) Output voltage stability test, reproduced under terms of the CC‐BY license [74]. Copyright 2023, Wiley‐VCH. (c) (i) Photocurrent at different wavelengths of the devices based on 2D IAO nanosheets with a bias voltage of 0.5 V. (ii) Photocurrent as a function of different power intensities. (iii) Multiple on/off photocurrent cycles. (iv) Single on/off photocurrent, reproduced with permission [40]. Copyright 2022, Wiley‐VCH. (d) Schematic diagram of the optoelectronic performance device. (i) Photocurrent at different wavelengths for a single layer of 0.1 at% Bi‐doped SnS. (ii) Photocurrent (*D*_Iph_) at different wavelengths with a bias voltage of 0.5 V. (iii) The response time at a wavelength of 285 nm and a frequency of 500 Hz measured between 10% and 90% of the step height. (iv) The photocurrent changes by ratio obtained at different wavelengths at *P*_inc_ = 2 mW cm^−2^ and *V*_ds_ = 0.5 V, reproduced with permission [107]. Copyright 2022, Royal Society of Chemistry.

A representative example is zinc–tin composite oxide, in which the enhanced sensing performance of zinc–tin composite oxide is attributed to the combined effect of the formation of a depleted layer at the surface of individual oxides (SnO_2_ and ZnO) as well as the formation of a hetero‐interface between these two oxides (Figure 8a) [31]. The mismatch in crystal lattice parameters between the 2D crystals also leads to additional defect states or oxygen vacancies, thereby enhancing oxygen adsorption on the surface and significantly improving the sensing performance [13, 31, 124]. In reducing gas sensing (e.g., NH_3_, H_2_), oxygen vacancies enhance oxygen chemisorption and accelerate reactions with target gases, leading to larger resistance changes and faster response–recovery kinetics due to reduced activation barriers. DFT studies further indicate that oxygen vacancies can increase adsorption energy and promote efficient charge transfer during gas adsorption and reaction processes [80].

### 5.2 Piezoelectricity and Photodetection

In principle, piezoelectricity arises in materials that lack a centrosymmetric crystal structure, where mechanical deformation generates an electric dipole moment. The introduction of point defects such as oxygen vacancies, interstitials, or substitutional dopants can further break local symmetry even within a nominally symmetric lattice. These defects create localized distortions and asymmetric charge distributions, resulting in enhanced or newly induced dipole moments. In defect‐engineered or mixed oxides, such local structural perturbations amplify spontaneous polarization and improve the coupling between mechanical strain and electrical response, thereby strengthening the overall piezoresponse. Guo et al. showed vacancy engineering from Bi_2_O_2.98_ to 2D Bi_2_O_2.68_ increased the *d*_33_and *d*_31_piezoresponses by approximately 650‐ and 210‐fold, respectively. (Figure 8b) [74]. This symmetry breaking was attributed to the rapid, non‐equilibrium instant‐printing process, which transfers the oxide before structural equilibration is reached. This asymmetry breaking was produced due to the instant‐printing process of the 2D MOs disrupting the growth by rapid transfer of the oxide before equilibrium had been reached [74]. However, excessive defects or dopants can disrupt the crystal structure and reduce the piezoelectric response. Therefore, careful control of the synthesis and defect‐engineering process is essential to optimize piezoelectric performance [74].

More recently, atomically thin substoichiometric β‐Ga_2_O_3−δ_ derived from liquid Ga exhibited an effective out‐of‐plane electromechanical response of approximately 4–5 pm V^−^^1^, despite the centrosymmetric lattice. The response was associated with defect‐mediated local symmetry breaking, supported by oxygen‐deficient XPS signatures, demonstrating that vacancy‐rich LM‐derived oxides can acquire electromechanical functionality absent from the ideal bulk crystal [104].

In photodetectors, trap states can strongly influence carrier recombination and photoconductive response. Oxygen‐related trap states can suppress charge‐carrier recombination and prolong photocarrier lifetimes, resulting in high photoconductive gain [125]. However, enhanced responsivity is often accompanied by slower response in metal‐oxide photodetectors [110, 125]. Defect engineering therefore requires control of carrier trapping to balance photoconductive gain and temporal response.

In LM‐derived materials, defect and dopant control has been used to tune electronic and optoelectronic properties. Antimony (Sb) doping was investigated in LM‐derived 2D In_2_O_3_ nanosheets at two doping levels: ∼1 at% (LD‐IAO, low‐doped) and ∼5 at% (HD‐IAO, highly doped) (Figure 8c) [40]. Both compositions exhibited excellent electronic and optoelectronic performance, comparable or superior to conventional 2D oxides and chalcogenides. The modulation of carrier density through Sb incorporation effectively tuned the Fermi level and enhanced electron mobility, demonstrating the potential of defect and dopant control for high‐performance devices [40].

Beyond oxide systems, defect‐mediated functional behavior is also increasingly relevant to LM‐assisted non‐oxide 2D materials, including chalcogenides and nitrides. Another notable example is 2D SnS synthesized from a Bi/Sn/In alloy under an H_2_S atmosphere through a selective migration mechanism (Figure 8d) [107]. Defects and spatially isolated potential barriers prolonged carrier recombination, resulting in response times several orders of magnitude longer than pristine SnS [107, 114]. The extended carrier lifetime is particularly promising for neuromorphic applications and highlights the potential of LM‐derived synthesis for tuning the optoelectronic properties of 2D materials [115].

### 5.3 Memory, Neuromorphic and Other Applications

In resistive‐switching and neuromorphic devices, mobile ionic defects, particularly oxygen vacancies, can directly participate in the formation and dissolution of conductive pathways [126, 127]. Under an applied electric field, oxygen‐vacancy migration and redistribution can locally modify the oxide reduction state and conductivity, enabling transitions between high‐ and low‐resistance states [126, 127]. The concentration, migration, and spatial distribution of these defects can therefore influence key switching parameters, including forming voltage, switching voltage, resistance level, endurance, and retention [127]. Higher oxygen‐vacancy concentrations can reduce switching voltages, whereas nonuniform vacancy distributions can produce different filament paths and increase device‐to‐device variability [127].

**FIGURE 9:**
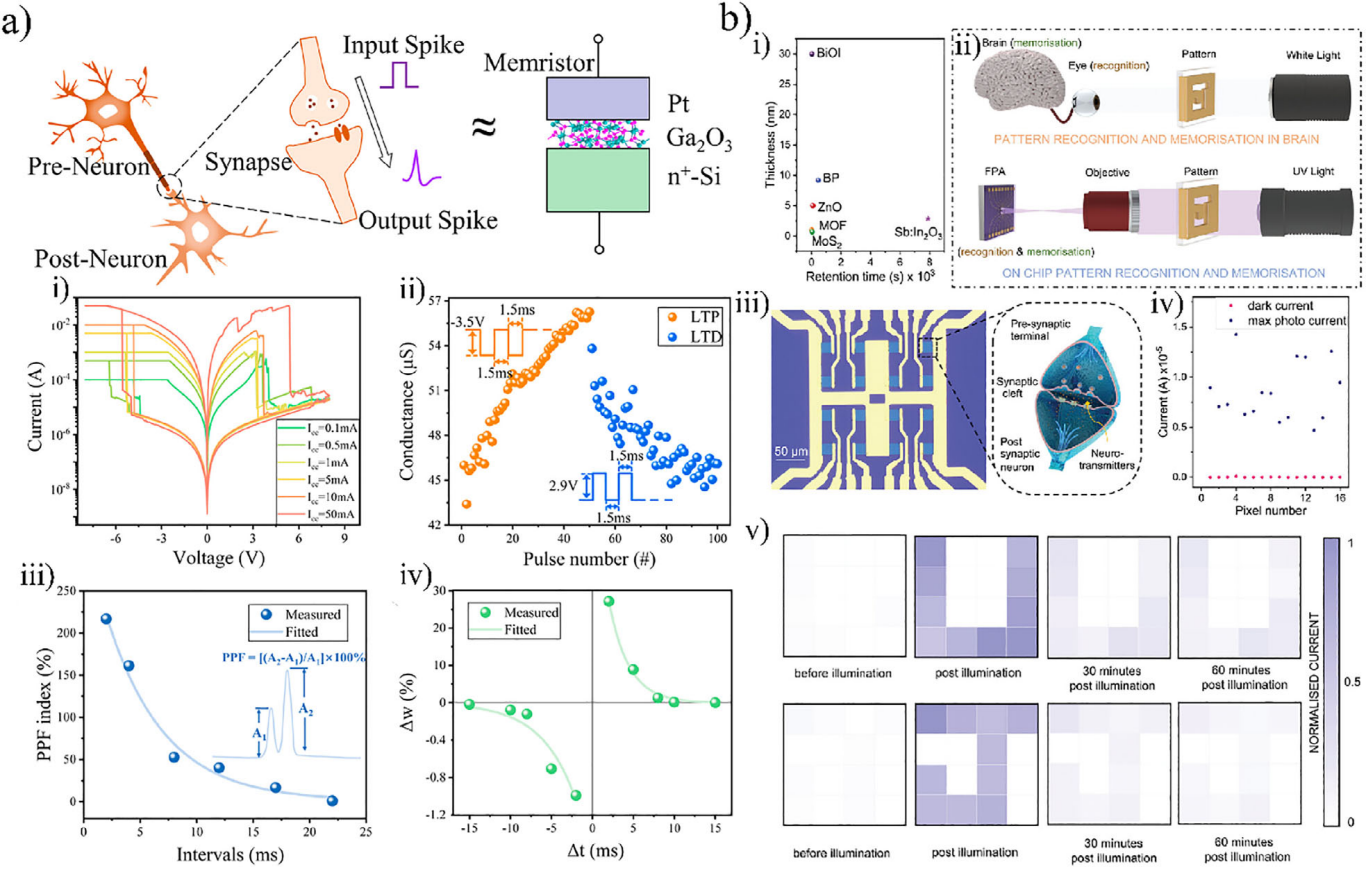
(a) Schematic diagram of a biological synapse (left) and two‐terminal Pt/Ga_2_O_3_/n^+^Si memristor (right). (i) Typical *I*–*V* curves of Pt/Ga_2_O_3_/n^+^Si memristors with different *I* ^+^_cc_. (ii) Long‐term potentiation (LTP) and depression (LTD) features mimicked by the artificial synapse of the Pt/Ga_2_O_3_/n^+^Si memristor. (iii) Paired‐pulse facilitation (PPF) index vs. interval between two consecutive pulses. Inset was the change in postsynaptic current stimulated by a pair of input sequences. (iv) Conductance (weight) change of the Pt/Ga_2_O_3_/n^+^‐Si memristor with a variation in Δ*t* shows the spiking timing‐dependent plasticity (STDP) characteristic, reproduced with permission [128]. Copyright 2023, American Chemical Society. (b) (i) Thickness and retention time of Sb:In_2_O_3_ photoconductors under ambient conditions with other reported synaptic photoconductors. (ii) Schematic representation of pattern recognition and memorization in the visual system and in the FPA. (iii) Optical image of a 4 × 4 pixel array of Sb:In_2_O_3_ devices, each device acts as an optoelectronic synapse. (iv) Graphical representation of the dark current and maximum photocurrent of all 16 pixels in the FPA. (v) Illustration of image memorization and prolonged retention by photocurrent generation using Letter patterns U and J under 3 mW cm^−2^ illumination using a 280 nm light source and 120 training pulses, reproduced with permission [133]. Copyright 2023, Wiley‐VCH.

LM‐printed Ga_2_O_3_ nanosheets, for example, exhibit stable resistive switching in Pt/ Ga_2_O_3_/n^+^Si memristors (Figure 9a), driven by oxygen‐vacancy‐based conductive filaments [128, 129, 130]. These devices successfully emulated key synaptic plasticity behaviors, stable switching, and synaptic functionalities such as long‐term potentiation, depression, and spike‐timing‐dependent plasticity. The defect‐mediated switching behavior of LM‐printed Ga_2_O_3_ nanosheets demonstrates the potential of defect‐engineered LM oxides for neuromorphic computing applications.

In optoelectronic neuromorphic devices, defect states can control the trapping and release of photogenerated carriers, thus controlling the magnitude and persistence of the photoresponse [131]. Repeated optical stimulation before complete carrier detrapping can extend the photocurrent decay process and promote the transition from short‐term to long‐term memory [131]. Persistent photoconductivity associated with oxygen‐vacancy‐related carrier dynamics can therefore enable synaptic functions such as potentiation and neural facilitation [132]. The trapping and release kinetics of defect states influence synaptic weight modulation, retention, and response dynamics, while long‐lived trapped carriers can result in slow recovery and persistent residual conductance [131, 132].

In liquid‐metal‐derived 2D materials, Sb‐doped In_2_O_3_ devices provide an example of this defect‐mediated mechanism. These devices exhibit optoelectronic synaptic behavior with reduced training cycles due to persistent photoconductivity (PPC) (Figure 9b) [133]. Under low bias, they demonstrate light‐induced synaptic functions, including multi‐synaptic responses, enabling in‐sensor neuromorphic computation. The observed frequency‐dependent learning behavior arises from defect‐induced trap states associated with dopants, oxygen vacancies, and ultrathin film thickness [134, 135, 136]. Such defect‐mediated processes highlight the potential of engineered 2D oxides for high‐performance optoelectronic neuromorphic systems [137].

Bandgap engineering represents another critical aspect of defect‐enabled functionality. By tuning alloy composition or dopant concentration at the liquid metal interface, the electronic structure and optical absorption of ultrathin oxides can be precisely controlled. For example, β‐(Al_x_Ga_1‐x_)_2_O_3_ nanosheets exhibit tunable bandgaps from 4.50 to 6.41 eV with increasing Al content, enabling deep‐UV emission and broadband photodetection [138, 139, 140, 141, 142, 143]. Similarly, Bi_2_O_3_‐doped indium tin oxide films show bandgap narrowing with increasing Bi concentration, enhancing optical absorption and charge transport in optoelectronic devices [16]. These examples demonstrate how programmable defect landscapes enable tunable electronic and optical functionalities in LM‐derived systems.

## 6 Future Directions in Programmable Defect Engineering

Despite rapid progress, defect engineering in LM‐derived 2D materials is still developing, and much of the control remains empirical, with limited ability to predict or reproducibly control defect populations. This limitation arises from a fundamental gap: defect formation is governed by the coupled interplay of interfacial redox chemistry, liquid‐phase transport, and mechanical deformation during growth and transfer, yet these processes are almost always studied in isolation. As a result, reported defect structures are highly system‐specific and not easy to reproduce, hindering both mechanistic understanding and technological translation. Addressing this challenge requires a shift from descriptive studies toward integrated, process‐level control of defect formation. Three priorities are particularly important: establishing quantitative synthesis‐defect relationships, directly observing defect formation and evolution under in situ or operando conditions, and achieving scalable control over defect type, density, and spatial distribution.

The establishment of quantitative and standardized descriptors for defect populations in liquid‐metal systems is also very important. Current reporting is fragmented, with defect density, type, and spatial distribution rarely measured under comparable conditions. Without consistent metrics, cross‐study comparisons remain unreliable, obscuring fundamental trends. We therefore call for the adoption of unified reporting protocols that link synthesis conditions (e.g., temperature, oxygen partial pressure, alloy composition, deformation rate) to statistically meaningful defect descriptors obtained from multiple characterizations. Such standardization will be essential to transition the field from qualitative observation to predictive design.

Another key priority is the development of operando characterization techniques that can capture defect formation at dynamic liquid interfaces. Most current insights rely on post‐transfer analysis, which cannot disentangle intrinsic growth‐induced defects from artefacts introduced during exfoliation and handling. Advancing in situ and operando approaches, such as spectroscopy, electron microscopy, and surface‐sensitive probes under controlled atmospheres, will be critical to directly observe defect nucleation, evolution, and healing in real time. This capability is particularly important for liquid‐metal systems, where rapid surface renewal and metastable states define the final structure of the exfoliated 2D materials.

A third major challenge is translating defect control from small‐scale experimental demonstrations to scalable and reproducible processing. This will require decoupled control over oxidation, fluid dynamics, and mechanical transfer, which currently act simultaneously and competitively. Strategies such as controlled interfacial confinement, programmable alloy compositions, and automated printing platforms offer promising routes to independently tune these parameters. Computational methods can further support translation by predicting defect stability, diffusion, and processing–structure relationships, while data‐driven approaches may accelerate defect identification and process optimization using large characterization datasets. Finally, the field must move beyond viewing defects as either beneficial or detrimental and toward programmable defect architectures with controlled spatial and functional characteristics. This includes engineering gradients, periodic defect arrays, and dynamic defect states that respond to external stimuli. Achieving this vision will require a clear understanding of how engineered defects influence device performance and reliability, including charge transport, leakage current, dielectric breakdown, and long‐term stability. Overall, integrating quantitative synthesis–defect relationships, in situ/operando characterization, and scalable processing will be central to establishing LM‐derived 2D materials as a platform for programmable defect engineering.

## 7 Conclusions

LM‐derived 2D materials offer a unique way to create ultrathin structures, where the liquid–solid interface plays an active role in material formation. In these systems, defects are naturally formed during growth and are closely linked to interfacial reactions, alloy composition, and processing conditions. Rather than being unwanted, these defects can be useful, as they strongly influence the electronic, chemical, and sensing properties of the materials.

A distinctive opportunity offered by liquid‐metal interfaces is that composition, doping, and defect formation can be coupled directly to crystal growth. Alloy constituents or deliberately introduced elements can selectively migrate to the liquid–gas interface and become incorporated into the growing lattice, enabling substitutional doping, mixed‐crystal formation, and heterointerface engineering without relying exclusively on post‐synthetic implantation or other disruptive processing. The asymmetric growth environment further enables spatial variations in composition and stoichiometry. For example, through‐thickness Ga‐rich and O‐rich regions have been resolved in ultrathin GaO_x_ using XRR and ToF‐SIMS, while atmosphere‐dependent phase and stoichiometry control has been demonstrated in LM‐derived tin oxides. Together with vacancy‐mediated symmetry breaking and controlled substoichiometry, these features establish liquid‐metal interfaces as a platform for encoding functional asymmetry directly during material formation. Such defect engineering already underpins applications in sensing, electromechanical response, optoelectronics, resistive switching, neuromorphic devices, and catalysis. More broadly, controlled coordination asymmetry, defect gradients, and metastable structures may provide opportunities in nonlinear optics and quantum light emission, solid‐state ion transport, and spin‐dependent phenomena, although these directions remain largely unexplored in LM‐derived 2D materials.

A major remaining limitation is the incomplete understanding of atomic‐scale and depth‐dependent defect structures. Direct evidence for the lattice positions of individual dopants and adatoms remains limited, with relatively few studies employing aberration‐corrected electron microscopy for this purpose. Likewise, the compositional gradients revealed by XRR and ToF‐SIMS in ultrathin GaO_x_ should be investigated systematically across other alloys and material systems. Complementary approaches such as XAS/XANES/EXAFS can resolve oxidation state, local coordination, and bond distortions, while atom‐probe methods may offer 3D compositional information where suitable sample preparation is feasible. This challenge is particularly important because freshly formed LM‐derived layers can rapidly oxidize, relax, or reconstruct after transfer; conventional ex situ measurements may therefore overlook metastable intermediates and the substoichiometric structures present immediately after growth. Rapid‐transfer, in situ, and operando characterization will be essential for resolving these transient states and establishing direct synthesis–defect relationships.

Translation to larger scales will additionally require control over materials utilization, film separation, reproducibility, and recovery or reuse of the parent liquid metal. These considerations are particularly relevant to catalysis, where the high surface‐to‐volume ratio of 2D materials can maximize the fraction of atoms exposed as functional active sites and potentially reduce the quantity of material required. However, this advantage can only be realized if active‐site density, defect distribution, layer separation, stability, and catalyst recovery are controlled reproducibly. Establishing these relationships will be critical for moving LM‐derived 2D materials from empirical synthesis toward predictive defect engineering and scalable functional technologies.

A key challenge for future research is to better control these defects. This will require a clearer understanding of how factors such as temperature, environment, and diffusion affect defect formation. The ability to stabilize different structures, including mixed phases and non‐equilibrium states, could open up new material functionalities. Improved characterization techniques, along with computational and data‐driven approaches, will help connect defect structure with performance. Overall, LM‐derived 2D materials provide a promising platform for designing functional materials for applications in sensing, electronics, and energy.

## Author Contributions

**Zhejun Hong**: conceptualization, investigation, writing – original draft, Writing – review and editing, visualization, validation, methodology, data curation, formal analysis. **Mohammad B. Ghasemian**: writing – review and editing, validation, investigation. **Chung Kim Nguyen**: conceptualization, data curation, validation, visualization, writing – review and editing. **Huy Hoang Nguyen**: validation, formal analysis, writing – review and editing. **Kourosh Kalantar‐Zadeh**: conceptualization, writing – review and editing, validation, methodology, formal analysis. **Torben Daeneke**: conceptualization, methodology, validation, writing – review and editing, formal analysis, supervision. **Nitu Syed**: supervision, conceptualization, investigation, methodology, validation, formal analysis, visualization, writing – original draft, writing – review and editing. **Ali Zavabeti**: supervision, conceptualization, investigation, funding acquisition, writing – original draft, writing – review and editing, validation, methodology, visualization, formal analysis.

## Ethical Statement

No ethical approval was required for this study.

## Conflicts of Interest

The authors declare no conflicts of interest.

## Data Availability

The data that support the findings of this study are available from the corresponding author upon reasonable request.
